# Risk assessment of mycotoxins, the identification and environmental influence on toxin-producing ability of *Alternaria alternate* in the main Tibetan Plateau *Triticeae* crops

**DOI:** 10.3389/fmicb.2022.1115592

**Published:** 2023-02-07

**Authors:** Jun Wang, Feilong Zhang, Ting Yao, Ying Li, Na Wei

**Affiliations:** ^1^Zhang Zhong-jing School of Chinese Medicine, Nanyang Institute of Technology, Nanyang, China; ^2^Institute of Agricultural Product Quality Standard and Testing Research, Tibet Academy of Agricultural and Animal Husbandry Sciences, Lhasa, China

**Keywords:** mycotoxin, *Alternaria alternate*, toxigenic capacity, hull-less barley, *Triticeae*

## Abstract

In order to find out the contamination of mycotoxins in *Triticeae* crops of Qinghai-Tibet Plateau, a total of 153 *Triticeae* crop fruits were collected as target samples, and 22 mycotoxins were tested. High detection rate was found in the *Alternaria* mycotoxins, including tentoxin (TEN), tenuazonic acid (TEA) and alternariol (AOH) toxins. To further clarify the production rules of *Alternaria* mycotoxins. A number of 9 high yield toxic strains were selected from 65 bacterial strains and the gene sequences of each were determined. The nine selected *Alternaria* alternate were cultured under specific pH of the culture medium, temperature and ultraviolet (UV) irradiation, and their growth and toxicity were analyzed. The results showed that the toxic capacity of most *A. alternate* increased with the increase of culture environment temperature and decreased with the increase of UV irradiation. However, the production of some toxins did not meet this principle, or even met the principle of relativity. In the culture experiments, a total of five *Alternaria* toxins were detected as positive, which were TEN, AOH, alternariol monomethyl ether (AME), TEA, and *Alternaria* (ALT). The altenusin (ALS) toxin was not detected in the metabolites of the nine *Alternaria* strains. It indicated that the TEN, AOH, AME, TEA, and ALT toxins should be particularly valued in the future risk assessments. This finding provided comprehensive information of mycotoxins contamination in the Tibetan Plateau *Triticeae* crops, it pointed out a direction to the Tibetan Plateau food crops’ quality control.

## Introduction

*Triticeae* crops, including hull-less barley (*Hordeum vulgare Linn.* var. nudum Hook. F.) and wheat (*Triticum aestivum* L.), were the most important food crops on the Tibetan Plateau for thousands of years (Shun et al., 2011; Galli et al., 2020) The quality and safety of *Triticeae* crops determined the Tibetan economy, people’s lives and health (Bryan et al., 2018). Both of hull-less barley and wheat had a long history of planting on the Qinghai-Tibet Plateau, with a large sown area and a wide adaptation range, from the valley slope below 2,500 m to the lakeside plain at around 4,200 m. Among the both, hull-less barley was the dominant characteristic crop of Tibet, and it was the basic ration crop on which the Tibetan people live survival and reproduction (Zeng et al., 2015). The planting area of hull-less barley accounted for about 70% of the total. Wheat was second only to hull-less barley in grain production in Tibet. It was planted in vast agricultural areas from humid and semi-humid areas to semi-arid and arid areas in western China (Cui et al., 2018). Therefore, the quality and safety of hull-less barley and wheat was directly related to the development of Tibetan agriculture and people’s lives (Chen et al., 2021).

The mycotoxins are a class of secondary metabolites with carcinogenic, teratogenic and mutational characteristics produced by fungi such as aspergillus and fusarium, which seriously threaten the health of human and animals (Pereira et al., 2014). At present, more than 400 kinds of mycotoxins had been found, among which the aflatoxin, ochratotoxin, and fumonisin et al. were the main mycotoxins that polluted the alimentarn crops, which could produce in the crop field growth stage to post-harvest storage stage (Piacentini et al., 2017; Hassan and Zhou, 2018). The pollution of food crops by mycotoxins was a widespread problem, and all countries in the world suffered from mycotoxin pollution to varying degrees. Statistical studies have found that the average annual economic loss of corn due to aflatoxin pollution in the United States was 1.68 billion dollars, and in Europe, the toxin pollution of deoxynivalenol was found in more than 44.6% of the tested grains (Qu et al., 2008; Ji et al., 2014; Mitchell et al., 2016). In China, the Ministry of Agriculture has carried out continuous monitoring of wheat mycotoxin pollution for many years and found that the mycotoxin in main wheat producing area was mainly contaminated by deoxynivalenol and zearalenone toxins (Qiu and Shi, 2014; Qiu et al., 2014). However, since the main *Triticeae* crops on the Qinghai-Tibet Plateau were hull-less barley, and the wheat planting area was limited, it did not belong to the main wheat producing areas in China, so the mycotoxin pollution of *Triticeae* crops on the Qinghai-Tibet Plateau was rarely reported.

Tibet had a special geographical location, diverse habitats and rich characteristic fungal resources, which was important value in scientific research. Differences in geographical environment and climatic conditions led to differences in microbial community composition in ecological zones. Natural environment had an important influence on the formation and evolution of biological species, and it was one of the hotspots of global biodiversity research (Fillinger et al., 2020). In China, systematic investigations and studies had been carried out on large fungal resources and characteristic resources in Tibet, and more than 2,000 fungal species have been found, among which endemic species to Tibet account for a high proportion. The altitude span of the agricultural production area in Tibet was 2,000 ~ 4,200 m (Li et al., 2015; Jiang et al., 2016). The geographical environment and climate factors such as temperature, rainfall and ultraviolet radiation changed with the altitude, thus forming complex environmental changes on the height gradient and affecting the species composition of microbial communities. Zhang et al. (2016) studied the impact of environmental conditions on soil microorganisms in Tibet and found that fertile and humid environment contributes to the growth of soil bacteria, and barren drought was conducive to the growth of soil fungi. Ren et al. (2018) studied microbial quantity, bacterial and fungal diversity and community composition with altitude gradient and found that high alpha diversity and the changes along the altitude ladder were greater than the fungal community, and soil temperature and humidity were the main causes of changes in bacterial community composition. However, few studies had been reported on the diversity, phylogeny and evolution of fungal fungi in the Tibetan Plateau.

In this work, in order to find out the contamination of mycotoxins in *Triticeae* crops of Qinghai-Tibet Plateau, 153 *Triticeae* crop fruits were collected as target samples, and 22 mycotoxins including aflatoxin B_1_ (AFB_1_), aflatoxin B_2_ (AFB_2_), aflatoxin G_1_ (AFG_1_), aflatoxin G_2_ (AFG_2_), ochratoxin A (OTA), ochratoxin B (OTB), ochratoxin C (OTC), sterigmatocystin (ST), zearalenone (ZEN), deoxynivalenol (DON), 3-Acetyl Deoxynivalenol (3-AcDON), 15-Acetyl Deoxynivalenol (15-AcDON), HT-2 toxin (HT-2), trichothecenes (T-2), fumonisin B_1_ (FB_1_), fumonisin B_2_ (FB_2_), alternariol monomethyl ether (AME), tenuazonic acid (TEA), alternariol (AOH), tentoxin (TEN), altenusin (ALS), and *Alternaria* (ALT) were tested in these samples. The 22 mycotoxins included *Aspergillus*, *Fusarium*, and *Alternaria* mycotoxins. These three classes of mycotoxins were widely detected in agricultural production (Agriopoulou et al., 2020; Li et al., 2021). The detection rates of TEN and TEA were the two highest targets, and all the two mycotoxins belong to *Alternaria* toxins (López et al., 2016; Mukesh et al., 2017; Gotthardt et al., 2019), produced by *Alternaria alternata* (Estiarte et al., 2016; Yang et al., 2017; Huang et al., 2020). In order to explore the mycotoxin production of *A. alternata*, a total of 65 bacterial strains were collected from different coordinate positions on the Qinghai-Tibet Plateau, then nine high yield toxic strains were selected and the gene sequences of each were determined. Tibet has a special geographical location, high altitude, strong ultraviolet light, and large temperature difference between day and night, which is bound to affect the toxic production of parasitic fungi in *Triticeae* crops on the Qinghai-Tibet Plateau (Liu et al., 2021; Yao et al., 2021). To clarify these rules, the nine selected bacterial strains were cultured under different experimental conditions, and their growth and toxicity were analyzed. The conclusion in this work was benefit for mycotoxin pollution reduction in *Triticeae* crops, scientific supervision and the Tibetan people’s consumption safety.

## Materials and methods

### Main reagents

A total of 153 crop fruits were obtained from Lhasa, Nyingchi, Qamdo, Shannan and Xigaze, and stored at 4°C. The standard solutions of 22 mycotoxins each containing 100 μg/mL were acquired from Pribolab Co. (Shandong, PR China), stored at-20°C. A total of 65 *A. alternata* were isolated from hull-less barley and wheat from different regions and stored at −80°C. The potato dextrose agar (PDA) medium was purchased from Qingdao Hope Bio-Technology Co., Ltd. (Shandong, PR China). Chromatographic grade of methanol, ammonium acetate, formic acid and acetonitrile were purchased from Merck Chemical (Shanghai, PR China).

### Detection of the mycotoxins in *Triticeae* crops samples

The 22 mycotoxins in the *Triticeae* crop fruits were analyzed by the standardized method with some modifications (Zhao et al., 2017).

### Sample pretreatment

The samples (5.0 g ± 0.01 g) were accurately weighed and placed into 50 mL centrifuge tube. The acetonitrile of 20 mL was added and a homogenization process was sustained for 30 min with a homogenizer to fully dissolve the mycotoxins into acetonitrile. Then the system was separated by a centrifuge at 6,000 rpm for 10 min. The supernatant of 1.5 mL was absorbed into a 5 mL plastic vial, and 0.5 mL of ultrapure water was added. After the system has been mixed evenly and filtered through a 0.22 μm PTEE, the solution was taken as sample solution for high-performance liquid chromatography - mass spectrometer (HPLC–MS/MS).

### HPLC–MS/MS analysis

An AB SCIEX Triple Quad™ 5,500 HPLC–MS/MS, with a CAPCELL PAK C_18_ MGIIfilled column (2.1 mm × 100 mm, 5 μm) was employed for separation and detection. The temperature of column for chromatographic separation was set at 35°C and the injection volume was set as 2.0 μl. The gradient elution program was set with mobile phase A of 0.1% formic acid (v/v) in 2 mol/l ammonium acetate and mobile phase B of methanol and a flow rate of 0.30 mL/min was set up. The modes of electrospray ionization (ESI) ion and multiple reaction monitoring (MRM) were used to determine the mycotoxins quantitatively. The ion spray voltage was set at 5500 V, the ion source temperature at 550°C and the curtain gas was 60 psi.

### Measurements of the growth parameters

The evaluation of *A. alternata* growth rate was performed by measuring the bacterial colony diameter of PDA medium. The medium was inoculated with 8 mm of *A. alternata* cake with the mycelial were faced downwards and cultivated in a special environment. The bacterial colony diameter was determined by cross method, and the diameter of bacterial colony was the diameter of the colony minus the diameter of the cake.

### Isolation of high toxin-producing ability of *Alternaria alternata*

In a sterile room, the toxin-producing ability of each *A. alternata* was determined by spread plated on the PDA medium and incubated at 25°C for 7 days. The PDA medium was scraped off the hyphae and dried at 60°C. The dried medium was blended with acetonitrile and placed for 12 h at room temperature. The mixture was filtered through a 0.22 μm PTEE to LC-MS/MS assay. According to the analytic results, a total of nine strains were selected from the 65 *A. alternata*, and the information of nine *A. alternata* was as shown in Table 1.

**Table 1:** The information of nine *Alternaria alternata.*

No.	Place of origin	Longitude	Latitude	Crop species	Date of acquisition	Altitude/m
S01-5	Jixiong Town, Gongga County, Shannan City	91.000813°	29.331504°	Wheat	2019	3,577
S05-5	Jixiong Town, Gongga County, Shannan City	90.993080°	29.297055°	Wheat	2018	3,574
2S10-1	Jixiong Town, Gongga County, Shannan City	91.017038°	29.28476°	Wheat	2018	3,588
R01-9	Langazi County, Xigaze City	90.467313°	29.142156°	Hull-less barley	2019	4,429
PL18-4b	Duoyou Village, Pulan Town, Pulan County, Ali District	81.164900°	30.324800°	Hull-less barley	2018	3,964
PL18-5b	Duoyou Village, Pulan Town, Pulan County, Ali District	81.164901°	30.324802°	Hull-less barley	2018	3,964
QBM02-13	Guxianggu village, Bomi County, Nyingchi City	95.470498°	29.907560°	Hull-less barley	2019	2,594
QBM05-10	Guxianggu village, Bomi County, Nyingchi City	95.470498°	29.907561°	Hull-less barley	2019	2,594
QBY06-2	Agriculture and Animal Husbandry College experimental site, Bayi District, Nyingchi City	94.340090°	29.672330°	Hull-less barley	2019	2,976

### Strain identification

The genomic DNA of the nine strains *A. alternata* was extracted using a GenEluteTM kit (Tiangen Biotech Co., Ltd., Beijing, China) in accordance with the manufacturer’s protocol. The PCR amplification was performed as follows: initial denaturation at 95°C for 3 min, 27 cycles of denaturing at 95°C for 30 s, annealing at 55°C for 30 s and extension at 72°C for 45 s. The 16S rDNA genes were amplified using the primers of 7F (5′-CAGAGTTTGAT CCTGGCT-3′) and 1540R (5′-AGGAGGTGATCCAGCCGCA-3′). PCR products were purified and sequenced by the Beijing Liuhe Huada Gene Technology Co., Ltd. After the alignment of the sequencing results on the NCBI website, the sequences with high homology were downloaded to construct the phylogenetic trees on the neighbour-joining method in MEGA 6.0 software.

### The effect of the *Alternaria alternata* growth conditions

#### The culture of nine toxigenic strains

In order to examine the toxin-production capacity of the nine toxigenic strains in different environments, the simulated environment of the Qinghai-Tibetan Plateau was constructed in the laboratory at an altitude of 3,650 m, and the activities of them were monitored in medium in relation to two different factors: the incubation temperature and the UV irradiation. Hyphal growth rate method was used to determine the effects of different conditions on fungi. The 15 mL culture medium was poured into 90 mm diameter dish. After cooling and solidification, a piece of 8 mm fungus cake was added in the center of each dish, with hyphae face down and placed in specific environmental species for culture, and each treatment was repeated three times.

### The detection

After 7 days of culture, the colony diameter was measured by the cross-crossing method, and the average value of the three measurements was used as the measurement result, minus the bacterial cake diameter as the colony diameter. The growing mycelia were carefully collected and dried at 60°C for 12 h. The toxin-production ability was measured by HPLC-MS/MS according to the proposed method. The targets were six common *Alternaria* toxins, including TEN, AOH, AME, TEA, ALS and ALT.

### The PH of the culture medium

The PDA culture medium was selected to verify the effect of pH conditions. The pH of PDA culture medium was adjusted with 0.2 mol/L disodium hydrogen orthophosphate to 5.8.

### Temperature

The nine strains *A. alternata* were individually inoculated on the PDA medium and incubated for at different temperature (15°C, 25°C, and 28°C) for 12 h, and then incubated at 10°C for 12 h, kept for 7 days. The diameter of colony was measured every other day since the third day. And toxin-production ability was evaluated according to the toxin-production after 7 days of culture.

### UV irradiation

The PDA medium was used to culture the nine strains *A. alternata* and exposed to with UV irradiation. After the inoculation of different strains, the medium was incubated at 25°C for 12 h, and then incubated at 10°C for 12 h, alternate ran kept for 7 days. From the third day on, the systems were irradiated for 0, 3, 5 h every 24 h 25°C. The effect of UV irradiation on the toxin production ability and growth of the strains were determined by measuring *Alternaria* toxins and bacterial colony diameter after 7 days.

### Statistical analysis

All data were presented as mean ± standard deviation and significance was accepted at *p* < 0.05. The Origin 2019 software (Origin Lab Co., Ltd.; USA) used to express the statistical data and graphs. One-way ANOVA followed by LSD were performed in SPSS 26.0 for Windows (SPSS Inc., Chicago, IL, USA) to determine the significance of the results of the toxigenic ability evaluation and the mycelial diameter.

## Results and discussion

### The analysis mycotoxins in 145 samples

**Figure 1:**
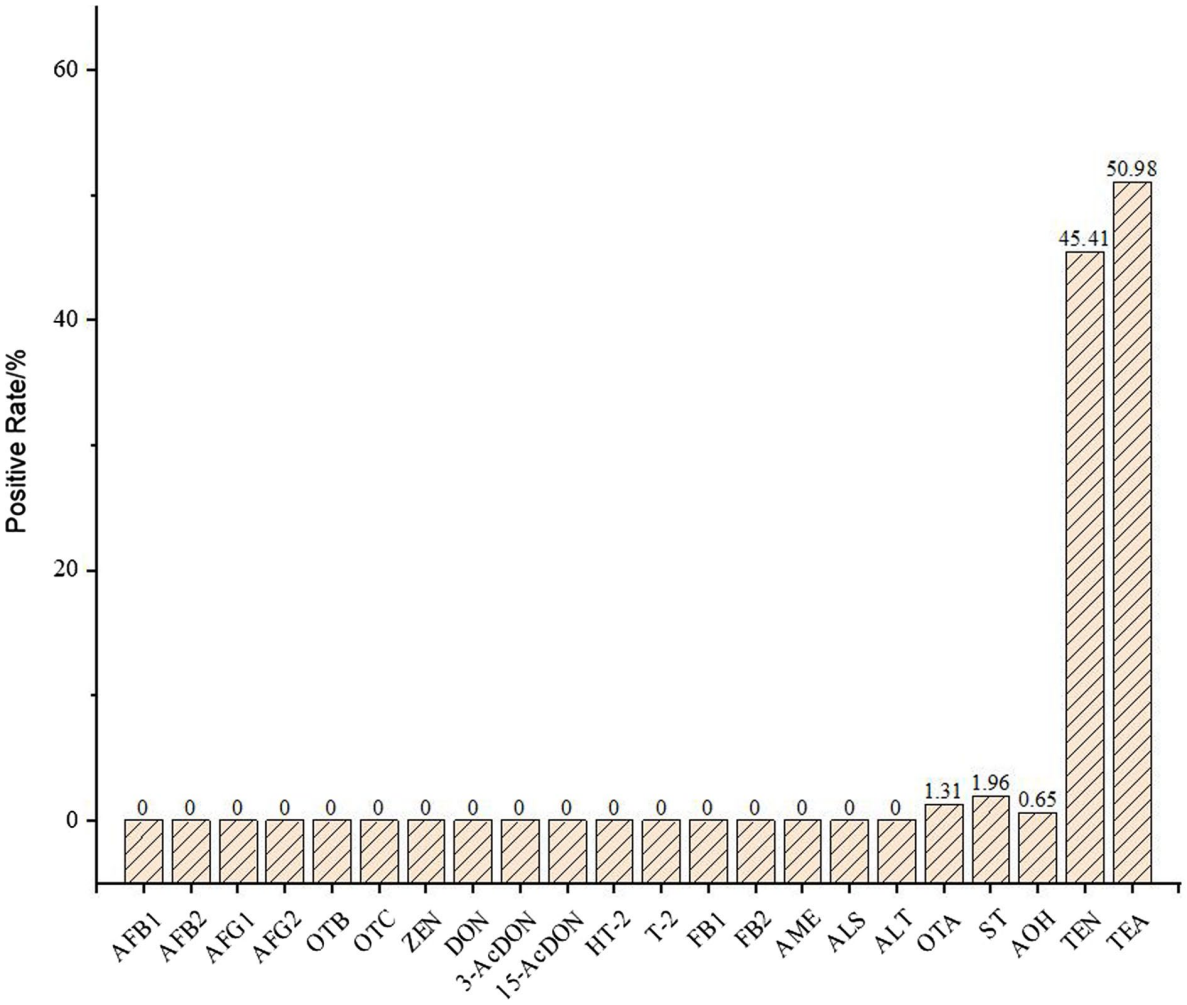
The positive rate of mycotoxins in 145 crop samples.

The mycotoxin contents in 153 hull-less barley and wheat samples were analyzed, the detection targets included AFB_1_, AFB_2_, AFG_1_, AFG_2_, OTA, OTB, OTC, ST, ZEN, DON, 3-AcDON, 15-AcDON, HT-2, T-2, FB1, FB2, AME, TeA, AOH, TEN, ALS, and ALT. According to the results, a total of 61 samples contained mycotoxins with overall positive rate was 39.87%, and 5 targets were detected, including TEN, ST, AOH, OTA, and TEA. As was shown in Figure 1, the positive rates of OTA, ST, AOH, TEN, and TEA were 1.31%, 1.96%, 0.65%, 45.41%, and 50.98%, respectively. The statistical results show that the positive rate of TEN and TEA even exceeded 45%, these two mycotoxins had a great risk of contamination of hull-less barley and wheat crops produced on the Tibetan Plateau. The TEN, TEA, and AOH of the five positive mycotoxins were all belonged to the *Alternaria* toxins, produced by *A. alternate*. This indicated that the *Alternaria* toxins should be monitored seriously in order to control mycotoxin contamination in food crops on the Tibetan Platea.

### The molecular biology identification

**Figure 2:**
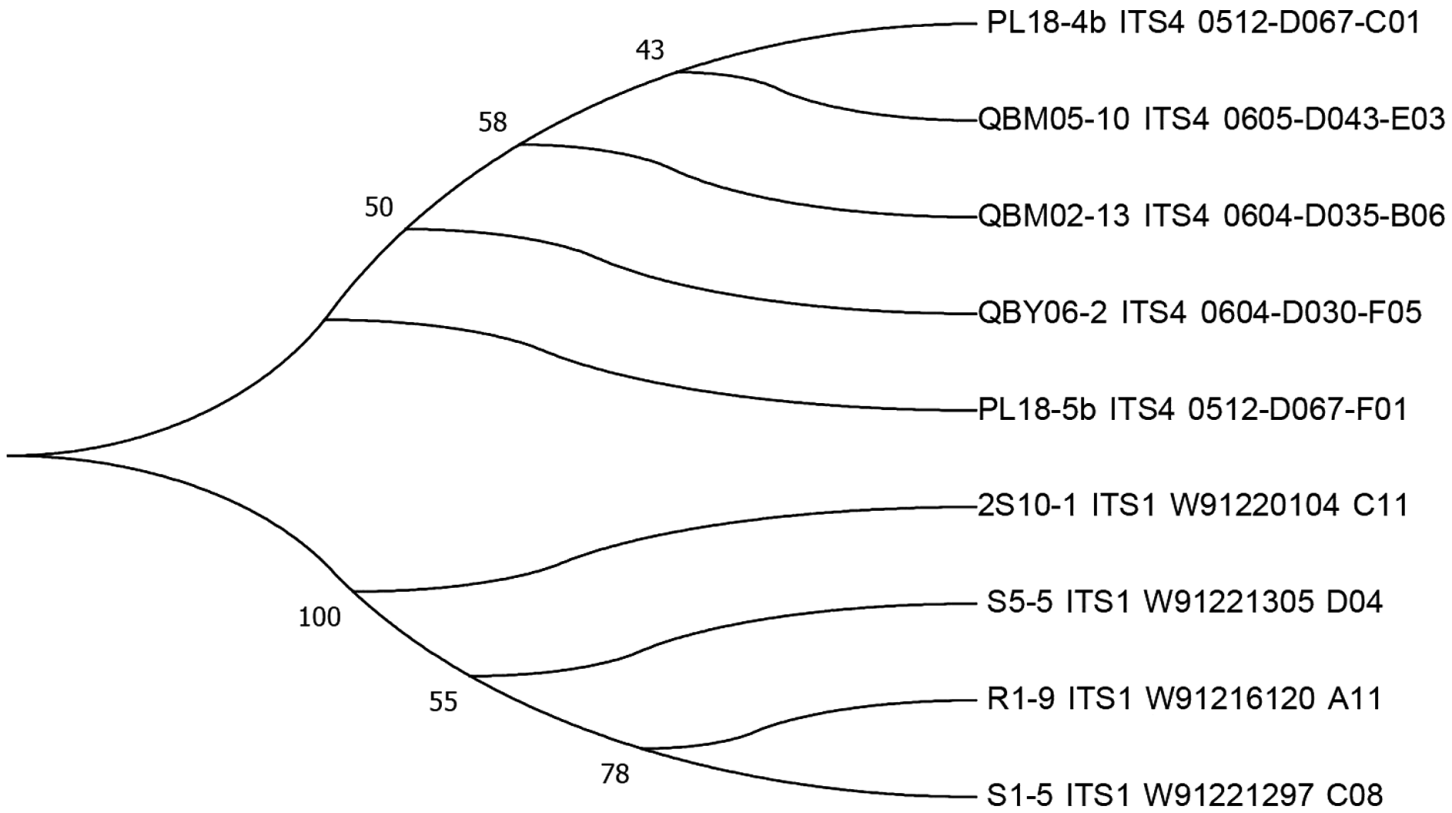
The phylogenetic trees of nine strains.

The sequencing results of the nine strains were uploaded to the GenBank data of the NCBI website for alignment, and the phylogenetic tree was constructed using MEGA 6.0 software, which was shown in the following Figure 2. The strains numbered PL18-4b, QBM05-10, QBM02-13, and QBY06-2 were developed from the same branch, which suggested that they were evolved from the same ancestor. The strains numbered 2S10-1, S05-5, R01-9, and S01-5 were developed from another branch, and the strain numbered 2S10-1 shared high homology with the other three strains. The strain numbered PL18-5b was from an independent branch, which indicated that this strain might display different toxigenic capacity properties compared to other two groups. In the subsequent test results, the ALT toxin was only detected in the metabolons of PL18-5b strain, while the other eight strains were not detected, which proved the particularity of PL18-5b to some extent.

### The selection of pH

The pH value of the origin environment had a great influence on the biological growth. Due to the presence of carbon dioxide in the air, the pH of the natural water environment was about 5.8, so the experiment was performed at pH 5.8.

### The effect of temperature on the toxigenic capacity

Temperature conditions played a crucial role in the life activities of strains. Studies on the growth and toxin-production capacity under different temperatures were important for controlling the fungal contamination of *Alternaria* toxin. Given the climatic conditions on the Tibetan Plateau, the temperature generally does not exceed 28°C, as much as possible to ensure that the highland barley and wheat could survive the normal growth period, so the three temperature conditions of 20°C, 25°C, and 28°C were selected for the experiment. Figure 2 showed the growth curves of nine *A. alternate* at different temperatures. As time went on, the results showed that all the nine *A. alternate* had the fastest growth rate at 28°C, with the second growth rate at 25°C and the lowest growth rate at 20°C.

**Figure 3:**
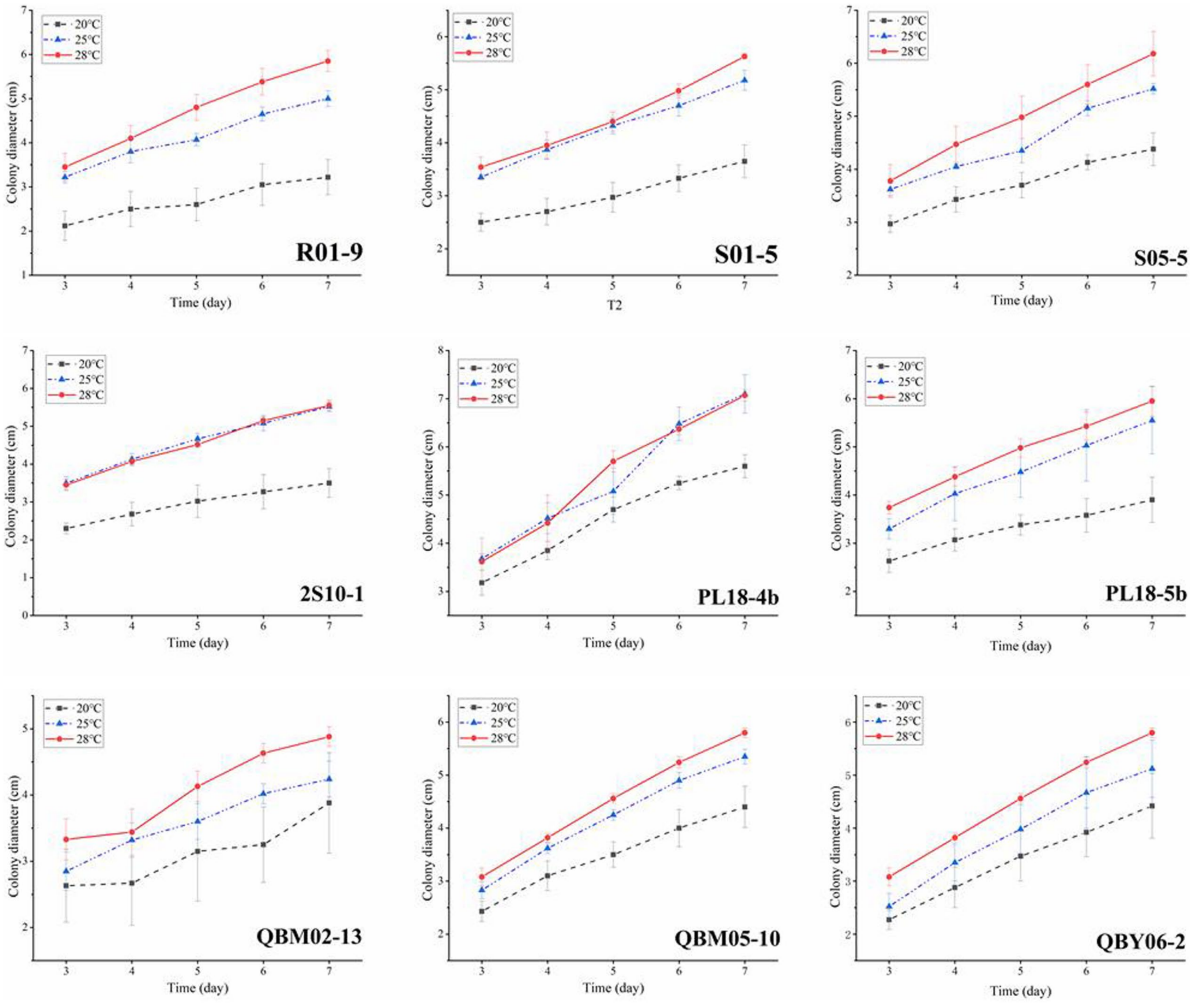
The effect of temperature on the growth of nine *Alternaria alternate.*

When the nine *A. alternate* were cultured for 7 days at 20°C, 25°C and 28°C, the *Alternaria* toxins in the culture medium was examined qualitatively and quantitatively, and the results were shown in Figure 3. As was shown in Figure 3, a total of five *Alternaria* toxins were detected as positive, which were TEN, AOH, AME, TEA, and ALT. The ALS toxin was not detected in the metabolites of the nine *Alternaria* strains. The TEN and TEA toxins were detected as high values among the metabolites of the nine *Alternaria* strains except for the strain numbered PL18-4b. This suggested that *A. alternate* had the strongest ability to produce both TEN and TEA toxins. The result could also explain the reasons of the high detection rates of TEN and TEA in the risk assessment to some extent. The AME toxin was detected as positive in eight *Alternaria* strains except for the strain numbered R01-9. This result indicated that the AME toxin has potential hazards in Tibetan crops, and sufficient attention should be paid to it in the future risk assessment. The AOH toxin was found in five *A. alternate*, which were numbered as S05-5, 2S10-1, PL18-4b, PL18-5b, QBM05-10, respectively. And the detected values of AOH toxin were relatively low. It was consistent with the low detection rate of AOH toxin in the risk assessment. The ALT toxin was detected in only one *A. alternate* numbered PL18-5b, and the detected values were very small. The ALS toxins were all negative in the metabolites of the nine *A. alternate*. Combined with the results of the risk assessment, it showed that the ALT and ALS toxins were less harmful than other 4 *Alternaria* toxins in food crops on the Tibetan Plateau.

**Figure 4:**
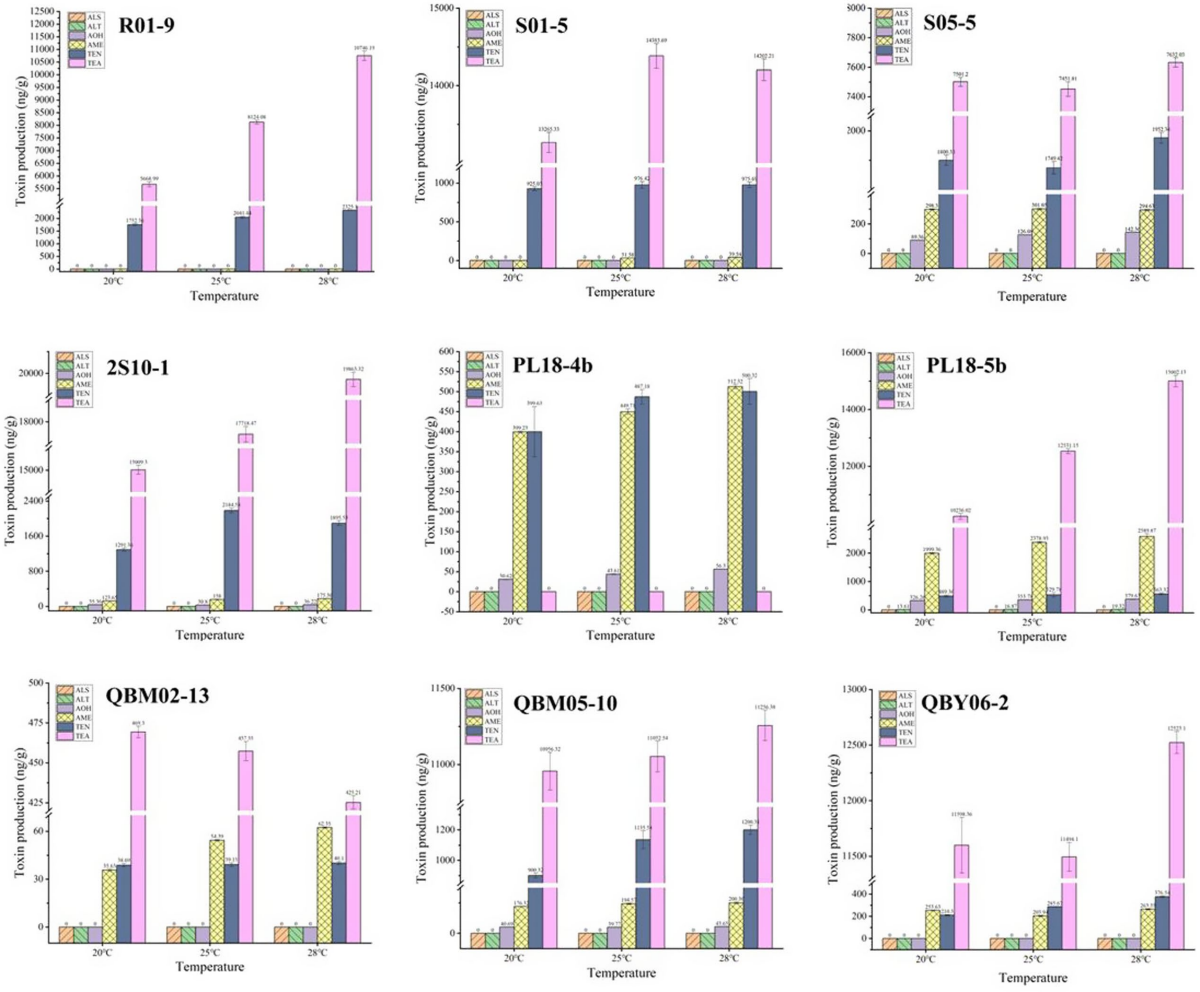
The toxin-production ability of nine *Alternaria alternate* at different temperatures.

After 7 days of incubation at 20°C, 25°C, and 28°C, according to Figure 4, a higher amount of toxins was produced at a higher temperature condition for most *Alternaria* toxins, such as the TEA toxins produced by the *A. alternate* numbered R01-9, 2S10-1, PL18-5b, QBM05-10, the TEN toxins produced by the *A. alternate* numbered R01-9, PL18-4b, QBM05-10, QBY06-2, and so on. This result was consistent with the growth of the nine *A. alternate* at different temperatures. However, the production of some toxins did not meet this principle, or even met the principle of relativity, such as the TEA toxins produced by the *A. alternate* numbered QBM02-13. Another phenomenon was found in the production process of many toxins, the production varied little at different culture temperatures, such as the TEN toxins produced by the *A. alternate* numbered R01-9, S01-5, QBM-02-13, the AME toxins produced by the *A. alternate* numbered S05-5, PL18-5b, QBM05-10 and so on. In conclusion, the law of toxicity production with culture temperature was diverse. Depending on the specific situation, the production of a single toxin by a single *A. alternate* was necessary.

### The effect of UV irradiation on the toxigenic capacity

**Figure 5:**
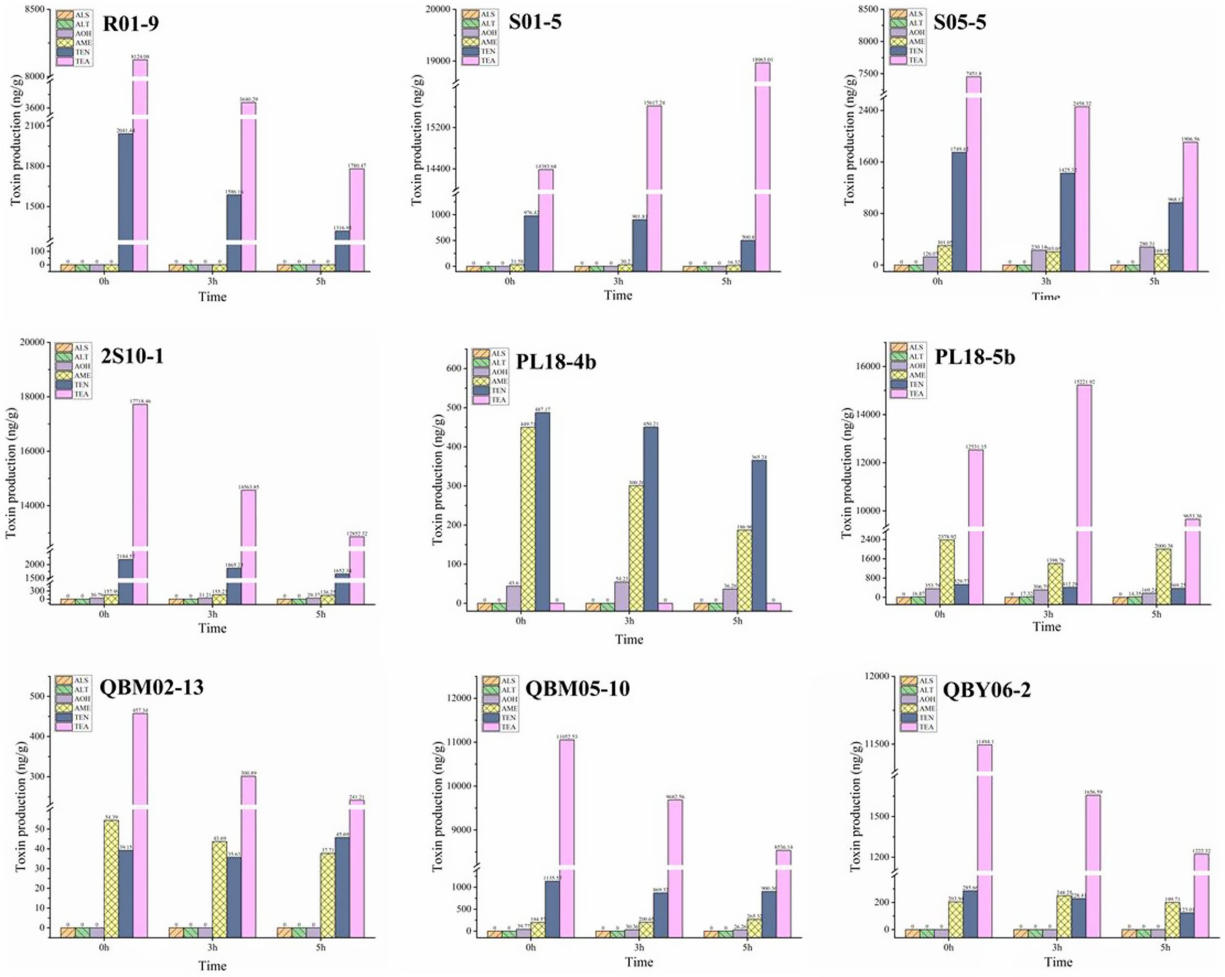
The effect of UV irradiation on the toxigenic capacity of nine *Alternaria alternate.*

During the 7-day culture period at 25°C, after 5 days of UV irradiation treatment under different duration, the detection results of *Alternaria* toxins produced in the culture medium were shown in Figure 5. According to the Figure 5, along with the prolonged time of UV irradiation, the trend of toxin production was decreased for most of nine strains. This suggested that the UV irradiation had suppressed the growth of nine strains, but a long duration or even 5 h could not completely kill the strains after 2 days of culture. When the growth of strain was inhibited, the toxin production was also adversely affected. However, the production of some toxins showed different phenomenon, or even presented the principle of relativity. The production of some toxins was not greatly affected by the UV irradiation, Such as the AOH toxins produced by the *A. alternate* numbered S05-5, 2S10-1, PL18-5b, the ALT toxins produced by the *A. alternate* numbered PL18-5b and so on. Moreover, a special phenomenon was discovered, such as the TEA toxins manufactured by the strain numbered S01-5, the toxin production increased with increasing UV irradiation time. When the UV irradiation intensity increased and the strain growth was inhibited, the toxigenic capacity increased. It showed that the production of toxins was not only related to the growth rate of the strains, but also related to the growth environment of the strains. In order to clarify the unclear rules, the subject should be further studied in the future.

## Conclusion

Hull-less barley and wheat were the most important *Triticeae* crops on the Tibetan Plateau. Prevention of mycotoxin contamination was of great significance for economic development and people’s health. In this study, mycotoxins in the main food crops on the Tibetan Plateau were monitored, and it was found that the main pollutants in the plateau food crops were *Alternaria* toxins, including TEN TEA and AOH. Subsequently, the 65 *A. alternate* were collected, and 9 strains were isolated and identified. The toxin-producing ability under different conditions was evaluated. According to the results, the selected nine *Alternaria alternate* could produce a variety of *Alternaria* toxins, which could not be ignored in terms of the quantity loss and quality reduction of plateau crops. The toxigenic capacity was not only related to the genetic constitution of the strains, but also related to the growth environment. It was necessary to keep monitoring and scientific evaluation of streptomycin in plateau crops, so as to effectively prevent the potential risk of contamination by streptomycin and other mycotoxins.

## Data availability statement

The data of 9 *Alternaria alternate* involved in this paper have been submitted and deposited in National Center for Biotechnology Information (https://www.ncbi.nlm.nih.gov/). The strain serial numbers and direct links can be found at: S01-5 - https://www.ncbi.nlm.nih.gov/nuccore/OQ347855, S05-5 - https://www.ncbi.nlm.nih.gov/nuccore/OQ347854, 2S10-1 - https://www.ncbi.nlm.nih.gov/nuccore/OQ347862, R01-9 - https://www.ncbi.nlm.nih.gov/nuccore/OQ347856, PL18-4b - https://www.ncbi.nlm.nih.gov/nuccore/OQ347861, PL18-5b - https://www.ncbi.nlm.nih.gov/nuccore/OQ347860, QBM02-13 - https://www.ncbi.nlm.nih.gov/nuccore/OQ347859, QBM05-10 - https://www.ncbi.nlm.nih.gov/nuccore/OQ347858, and QBY06-2 - https://www.ncbi.nlm.nih.gov/nuccore/OQ347857.

## Author contributions

JW and NW conceived and designed the experiments. FZ and TY performed the experiments. JW and NW supervised the project. FZ and YL analyzed the data. JW, NW, and FZ wrote the manuscript. All authors contributed to the article and approved the submitted version.

## Funding

This research was supported by National Natural Science Foundation of China (no. 31860473), The Project of the Science and Technology Department of Tibet, China (no. XZ202101ZY0015G), National Natural Science Foundation of Tibet, China (no. XZ202101ZR0067G) and The Doctoral scientific research launch fund project of Nanyang Institute of Technology (no. 510198).

## Conflict of interest

The authors declare that the research was conducted in the absence of any commercial or financial relationships that could be construed as a potential conflict of interest.

## Publisher’s note

All claims expressed in this article are solely those of the authors and do not necessarily represent those of their affiliated organizations, or those of the publisher, the editors and the reviewers. Any product that may be evaluated in this article, or claim that may be made by its manufacturer, is not guaranteed or endorsed by the publisher.
